# Final hard copy issue of the Scandinavian Journal of Primary Health Care – online only from 2014

**DOI:** 10.3109/02813432.2013.864089

**Published:** 2013-12

**Authors:** Peter Vedsted

**Affiliations:** Editor-in-Chief, Scandinavian Journal of Primary Health Care. E-mail: p.vedsted@alm.au.dk

Scandinavian Journal of Primary Health Care is a modern medical journal dedicated to publishing family medicine research. The journal seeks to be among the leading similar journals in the world. One of the great developments within publishing scientific papers has been the use of electronic media where it is possible to make an early and fast publication of new knowledge. It has become more and more evident that new knowledge should be easily accessible and available at a very early stage to facilitate early implementation of important medical knowledge. This implies an open and easy access to papers published in a medical journal.

Increasing medical knowledge demonstrates implications for not only clinical professionals, but also for the organisation and the performance of the health care system. This is seen e.g. by the growing amount of health services research within family medicine. Thus, patient-oriented clinical research, population-based epidemiological research and health care oriented health services research are now in high demand from clinicians, decision makers and researchers.

Scandinavian Journal of Primary Health Care plays an important role in disseminating such knowledge. Therefore, it is highly prioritised to ensure that research supported by public funding is also publicly available. During a number of years, Scandinavian Journal of Primary Health Care has provided open access to the papers published in the journal. All papers are freely available online and through PubMed. The Nordic Federation of General Practice is proud of such a substantial contribution and is now taking a further step in that development.

From January 2014, after 31 paper volumes since 1983, Scandinavian Journal of Primary Health Care will not be printed on paper. The journal will become an online-only medical journal benefiting from all the possibilities inherited with that. This also implies that there will not be a paper version of the Scandinavian Journal of Primary Health Care in your mailbox anymore. You now have to make sure that you, if not already, have signed up for an e-mail version of the journal using the link below.

With this change a fantastic and important era with a paper journal from the Nordic countries has ended. Nevertheless, the Scandinavian Journal of Primary Health Care will continue to develop into an even more important journal within family medicine and try its best to support patient care on a knowledge-based platform. This is only possible as the journal receives such a huge amount of relevant and well-done research.

## Other changes in 2014

As an online-only journal, Scandinavian Journal of Primary Health Care, will be allowed to publish in colour, publish additional online material like questionnaires and to accept longer manuscripts, if relevant. The layout of the papers will change slightly during 2014, and we look forward to presenting the new design. Lastly, the new online structure will give us all the full advantages of using our website and the social media.

Open access makes academic research in primary care freely available online. However, this also means that the journal has increased author fees. The author fees will be one for members of one of the Nordic societies for general practice, and one fee for non-members. For more details, please refer to the journal's website at www.sjphc.org.

